# Supplementary material for: Long-Term Sodium Deficiency Reduces Sodium Excretion but Impairs Renal Function and Increases Stone Formation in Hyperoxaluric Calcium Oxalate Rats
Source: Int J Mol Sci. 2024 Apr 1;25(7):3942. doi: 10.3390/ijms25073942 (PMC11011831; doi:10.3390/ijms25073942)
Supplement: Supplementary file 1 [file ijms-25-03942-s001.zip › Table S1.pdf]

Supplementary Table S1. Renal function and AP (CaOx) index data from short-term sodium balance study.

Groups / Parameters	Control	EG	*P* value
Day 42 after 5-day no sodium diet			
Water intake, ml/day	37.6 ± 8.9	54.2 ± 4.8*	<0.001
Urine amount, ml/day	35.9 ± 9.2	44.5 ± 4.4*	0.001
Urine protein, mg/day	23.5 ± 6.8	17.4 ± 5.9	0.731
C_{Cr} , ml/min/kg	1.6 ± 0.4	1.5 ± 0.4	0.181
Posm, mosmol/kg/H_2O	292.3 ± 4.7	333.2 ± 6.0*	<0.001
Uosm, mosmol/kg.H_2O	1252.3 ± 22.0	836.2 ± 28.9*	<0.001
U/Posm ratio	3.6 ± 0.1	2.5 ± 0.1*	<0.001
FE_{Na} (%)	0.08 ± 0.04	0.50 ± 0.31*	0.048
FE_k (%)	24.3 ± 3.0	42.0 ± 6.0*	0.018
U_{Na}×U_v, µmol/min/kg	0.3 ± 0.1	0.3 ± 0.0	0.534
U_K×U_v, µmol/min/kg	7.6 ± 0.8	5.0 ± 0.8	0.051
Urine Ca/Cr	0.3 ± 0.0	0.7 ± 0.3	0.073
T_cH_2O, µl/min/kg	72.8 ± 8.4	104.4 ± 6.5*	0.013
Urine oxalate, mg	6.2 ± 2.1	66.1 ± 4.5	<0.001
AP (CaOx) index	5.5 ± 1.0	25.0 ± 7.3	0.074

SD, sodium deficient diet ; EG, Ethylene glycol; p, plasma; Posm, plasma osmolality; Uosm, urine osmolality; FE_{Na}, fractional excretion of Na; U_{Na}×Uv , rate of urinary Na excretion; FE_K, fractional excretion of K; U_K×Uv, rate of urinary K excretion; T_cH_2O, solute-free water reabsorption; AP (CaOx) index, the ion activity product of CaOx. * p<0.05, compared to control group.
