# Supplementary figures and images for: Long-Term Sodium Deficiency Reduces Sodium Excretion but Impairs Renal Function and Increases Stone Formation in Hyperoxaluric Calcium Oxalate Rats
Source: Int J Mol Sci. 2024 Apr 1;25(7):3942. doi: 10.3390/ijms25073942 (PMC11011831; doi:10.3390/ijms25073942)

EG

SD+EG

H&E

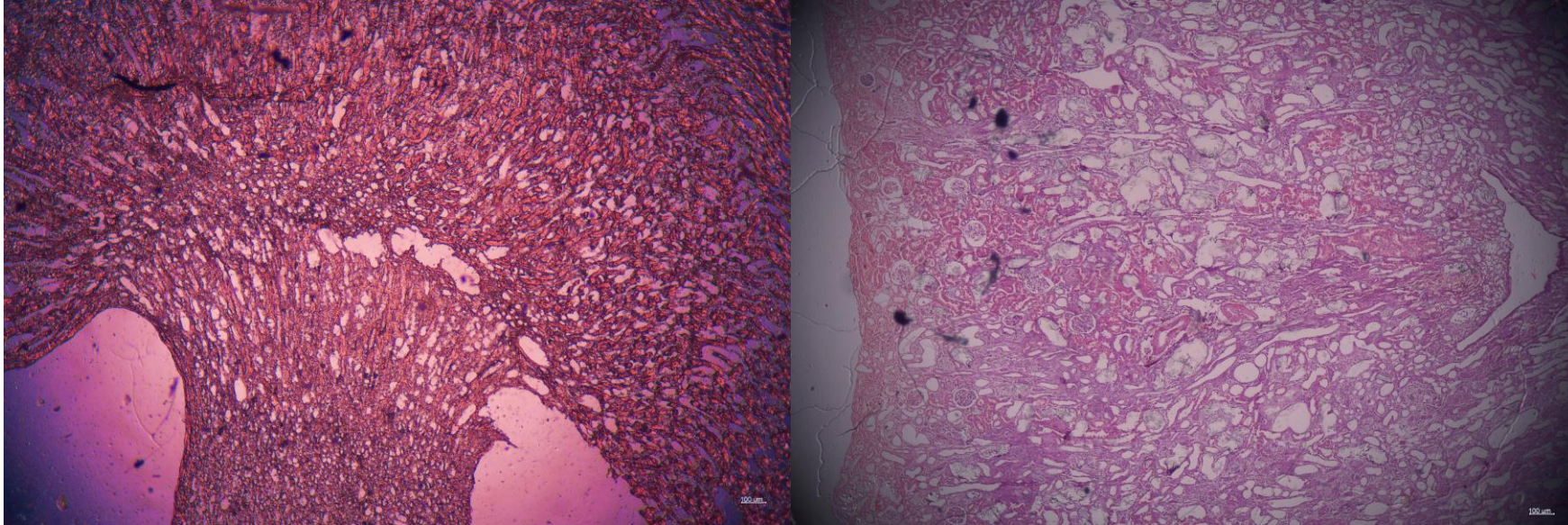

Polarizing  
microscope

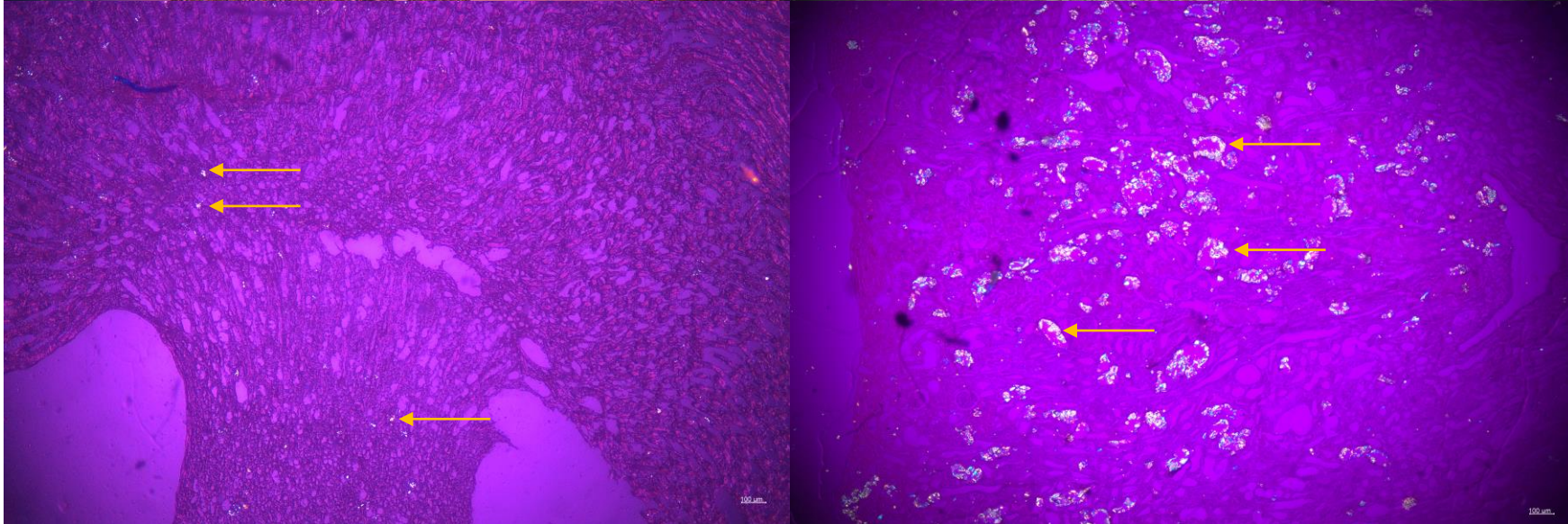

\*Yellow arrow: crystal deposition

Supplement: Supplementary file 1 [file ijms-25-03942-s001.zip › Figure S1.pdf]

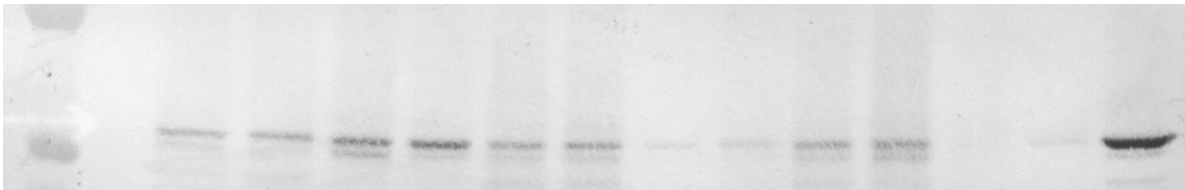

BSC-1

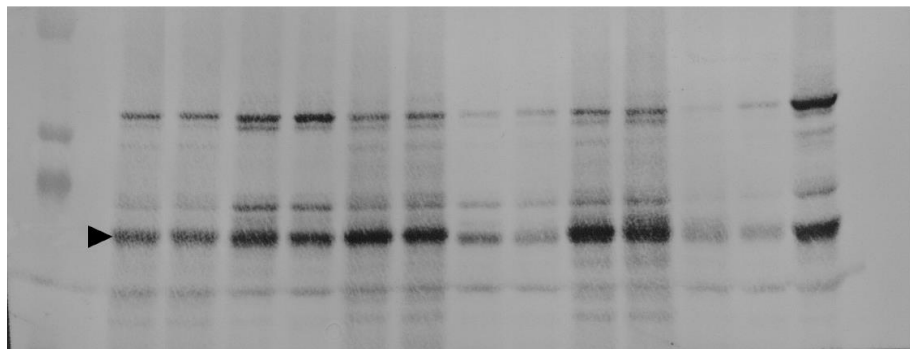

NHE3

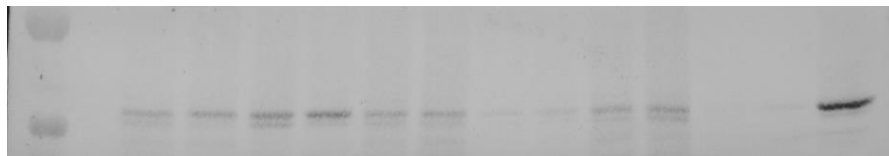

TSC

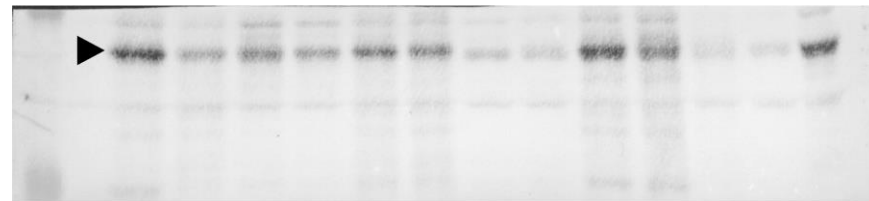

$\alpha$ -ENaC

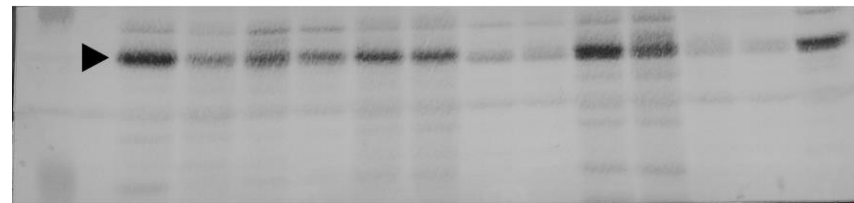

$\beta$ -ENaC

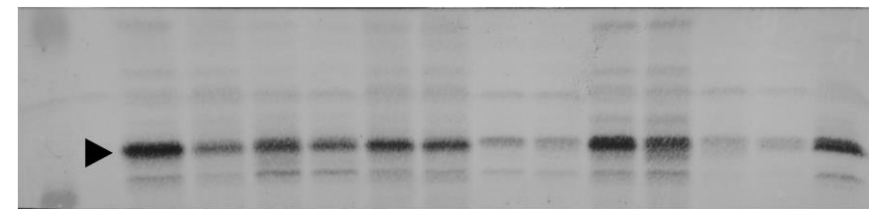

$\gamma$ -ENaC

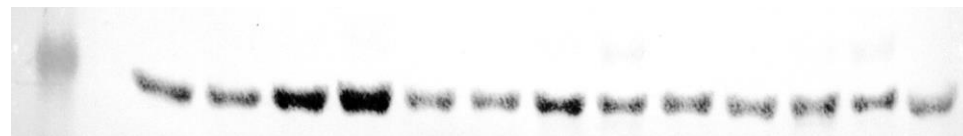

$\beta$ -actin

Supplement: Supplementary file 1 [file ijms-25-03942-s001.zip › Figure S2.pdf]

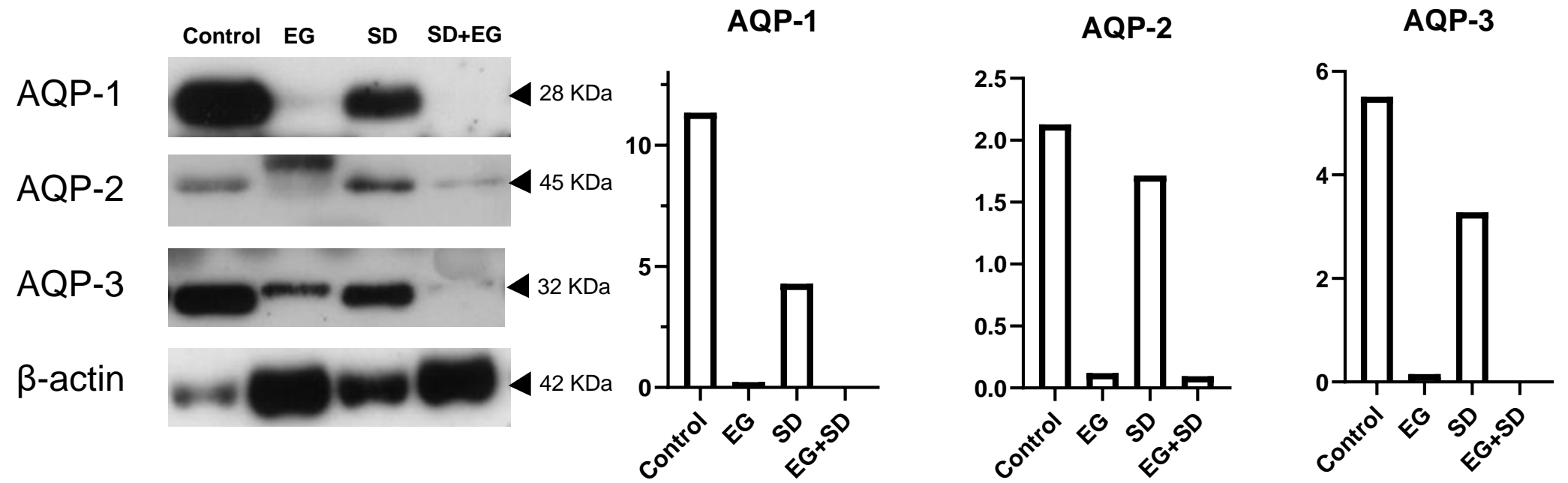

Supplement: Supplementary file 1 [file ijms-25-03942-s001.zip › Figure S3.pdf]
